# The chromatin scaffold protein SAFB1 localizes SUMO-1 to the promoters of ribosomal protein genes to facilitate transcription initiation and splicing

**DOI:** 10.1093/nar/gkv246

**Published:** 2015-03-23

**Authors:** Hui-wen Liu, Tapahsama Banerjee, Xiaoyan Guan, Michael A. Freitas, Jeffrey D. Parvin

**Affiliations:** 1 Department of Biomedical Informatics, Comprehensive Cancer Center, The Ohio State University, Columbus, OH 43210, USA; 2 Department of Molecular Virology, Immunology, and Medical Genetics, Comprehensive Cancer Center, The Ohio State University, Columbus, OH 43210, USA

## Abstract

Early steps of gene expression are a composite of promoter recognition, promoter activation, RNA synthesis and RNA processing, and it is known that SUMOylation, a post-translational modification, is involved in transcription regulation. We previously found that SUMO-1 marks chromatin at the proximal promoter regions of some of the most active housekeeping genes during interphase in human cells, but the SUMOylated targets on the chromatin remained unclear. In this study, we found that SUMO-1 marks the promoters of ribosomal protein genes via modification of the Scaffold Associated Factor B (SAFB) protein, and the SUMOylated SAFB stimulated both the binding of RNA polymerase to promoters and pre-mRNA splicing. Depletion of SAFB decreased RNA polymerase II binding to promoters and nuclear processing of the mRNA, though mRNA stability was not affected. This study reveals an unexpected role of SUMO-1 and SAFB in the stimulatory coupling of promoter binding, transcription initiation and RNA processing.

## INTRODUCTION

Small Ubiquitin-related Modifier (SUMO) proteins are highly conserved among eukaryotes, and protein SUMOylation has a critical role in a variety of cellular signaling pathways including control of cell cycle progression, DNA repair, gene expression and nuclear architecture (1). Among various SUMO substrates that have been identified, transcription factors and co-regulators comprise one of the largest groups. Studies have provided strong evidence for the involvement of SUMOylation in transcriptional regulation (2). SUMOylation of those transcription factors in general is repressive, and current models suggest that SUMOylation leads to the recruitment of transcriptional co-repressor complexes and histone deacetylases (HDACs) to the promoters (3,4). However, there is also evidence that SUMOylation of transcription factors can lead to gene activation (5–7). In a previous study, we found that SUMO-1 modifies chromatin-associated proteins located at the promoter regions of highly active genes in human cells, including those that encode ribosome protein subunits (8). SUMO association on active promoters has also been observed in yeast and in human fibroblasts (9,10). These studies have suggested that SUMOylation of transcription factors is not merely acting as a switch for gene silencing; rather, it also plays an important role for modulating transcription activation. However, the role of how SUMOylation modulates chromatin structure, and further participates in transcriptional control of constitutive genes is largely unknown.

In this study, we first sought to identify the SUMOylated protein bound to the chromatin at active promoters, and we found that Scaffold Associated Factor-B (SAFB), a DNA and RNA binding protein, is one of the SUMO-1 targets. Two homologs (SAFB1 and SAFB2) have been found with 74% similarity at the amino acid level, and up to 98% similarity in some functional domains and display redundant activity (11). SAFB1 interacts with the carboxy-terminus of RNA polymerase II (RNAPII) and RNA processing proteins such as SR proteins (12–15), suggesting a potential role in RNA splicing. SAFB binds AT-rich scaffold/matrix attachment regions (S/MAR) on DNA, which are found close to regulatory loci and mediate chromatin looping to coordinate distant chromatin interactions and higher order chromatin structure (16,17). SAFB proteins interact with RNA through the RNA recognizing motif (RRM), which suggests a role in mRNA processing. Together, this suggests that SAFB may be part of a ‘transcriptosome complex’ to couple transcription, splicing, and polyadenylation (13). This hypothesis is supported by a study that SAFB1 interacts with CHD1, a chromatin modifying protein that also possesses activities in RNA splicing (18,19). In addition, SAFB has been found to function as a co-repressor of estrogen-dependent transcription (20), and participates the repression of immune regulators and apoptotic genes (21). Recent studies suggest that it may be involved in a more widespread manner by functioning as a positive regulator for permissive chromatin of the myogenic differentiation (22), and in response to DNA damage (23).

Here, we provide evidence that both SAFB1 is a SUMO-1 substrate bound to the chromatin during interphase in a region centering on 100 bp upstream of the transcription start site. Like SUMO-1, depletion of SAFB diminished RNAPII binding to promoters and decreased RNA expression of these ribosomal protein genes, revealing an unexpected role of SAFB linking transcription initiation to RNA processing of the highly active ribosomal protein (RP) genes.

## MATERIALS AND METHODS

### Chromatin affinity purification (ChAP) for mass spectrometry analysis

ChAP was based on the ChIP method except that the immunoprecipitation was replaced by a two-step affinity purification from HeLa-SUMO1 cells, a HeLa-derived cell line that expresses a SUMO-1 protein that includes on its amino-terminus a hexa-histidine tag and a biotin binding domain (8). Cells were synchronized in S phase or in mitosis. 10^8^ HeLa-SUMO1 cells were lysed in lysis buffer I (50 mM 4-(2-hydroxyethyl)-1-piperazineethanesulfonic acid–potassium hydroxide (HEPES–KOH), pH 7.5, 140 mM NaCl, 1 mM Ethylenediaminetetraacetic acid (EDTA), 10% glycerol, 0.5% NP-40, 0.25% Triton-X-100), and the cell pellet was resuspended in lysis buffer II (10 mM Tris, pH 8, 80 mM NaCl, 1 mM EDTA, 0.5 mM ethylene glycol tetraacetic acid (EGTA)), and, following centrifugation (1400 x *g*), the chromatin was recovered from the pellet and resuspended in lysis buffer III (50 mM Tris pH 8; 0.01% SDS; 1.1% Triton X-100; 80 mM NaCl). The isolated chromatin was sheared to 200–300 bp by sonication, incubated with 375 μl of Ni-NTA beads (Qiagen) for 16 h at 4°C. After washing in wash buffer I (50 mM Tris pH 8; 0.01% SDS; 1.1% Triton X-100; 150 mM NaCl), chromatin fragments were eluted in 6 ml elution buffer (washing buffer I with 300 mM imidazole). The nickel eluate was incubated with 375 μl of streptavidin beads (Invitrogen) for 6 h at 4°C. After three stringent washes, wash buffer II (50 mM Tris pH 8; 10 mM EDTA; 1% SDS; 1M NaCl), and three times of 10 mM Tris buffer, pH 8. The chromatin was then trypsinized (ratio 1:120 w/w) in solution for 2 h at 37°C. Following lyophilization, the digested solution was dissolved in 50 μl high performance liquid chromatography (HPLC) grade water and subjected to LC–MS/MS; protein identification was analyzed using Massmatrix 2.4.2 (24). Environmental contaminants were removed from the identified proteins.

### Antibody used in this study

The rabbit polyclonal SUMO-1 and 8WG16 monoclonal antibodies were described previously (8,25). Other antibodies used included the RNAPII phospho-serine 5 antibody (Abcam cat. no. ab5131), SAFB antibody (Millipore cat. no. 05-588) and α-tubulin (Sigma).

### Isolation of nuclear and cytoplasmic RNA, RT-qPCR

Nuclear and cytoplasmic RNAs were purified by lysis of HeLa-SUMO1 cells in 200 μl of lysis buffer (10 mM Tris–HCl, pH 8, 1.5 mM MgCl_2_, 0.5% NP-40, 140 mM NaCl and 0.5 U of RNase inhibitor) and loaded onto 200 μl of cushion buffer (10 mM Tris–HCl, pH 8, 1.5 mM MgCl_2_, 1% NP-40, 140 mM NaCl and 0.4 M sucrose). The samples were centrifuged for 10 min at 800 × *g*. The cytoplasmic supernatant was treated with proteinase K, extracted with phenol–chloroform and precipitated with ethanol. The nuclear pellet was resuspended in 100 μl of DNAse I buffer (50 mM Tris–HCl, pH 7.5, 1 mM EDTA, 10 mM MgCl_2_ and 0.5 U RNase inhibitor) and treated 20 U of DNase I (Invitrogen) for 60 min at 37°C. The samples were then extracted with phenol–chloroform and precipitated with ethanol. The mRNA levels were determined by reverse transcriptase quantitative polymerase chain reaction (RT-qPCR) using the iScript reverse transcription and iQSYBR Green supermix (Bio-Rad). The results were normalized to 18S rRNA.

### Immunoprecipitation, ChAP-qPCR and ChIP-qPCR

Immunoprecipitation was performed following established procedures (26). Chromatin immunoprecipitation or affinity purification was done following the protocol that has been reported previously (8). Briefly, 2 × 10^7^ HeLa cells (if ChIP) or HeLa-SUMO1 cells (if ChAP) were fixed with 1% formaldehyde, lysed in lysis buffer, sonicated to size ranging from 200 to 1000 bp, chromatin was diluted 4-fold, after removal of a control aliquot, incubated at 4°C overnight with antibody against 8WG16 (1:100), 5 μg Ser-5p antibodies, or 5 μg IgG antibody was used as a negative control. Immunocomplex was precipitated with 50 μl protein A sepharose beads (GE Healthcare), washed with Radioimmunoprecipitation assay buffer, 10 mM Tris pH 7.5, 300 mM NaCl, 1% NP-40, 1% deoxylcholate, 0.1% SDS (RIPA) buffer followed by crosslink reversal. The immunoprecipitated DNA was purified by PCR purification kit (Qiagen). All the experiments included at least three independent replicates. The primer sequences are available in Supplementary Table S2.

## RESULTS

### SUMOylation facilitates RNAPII recruitment on constitutively active promoters

**Figure 1.**
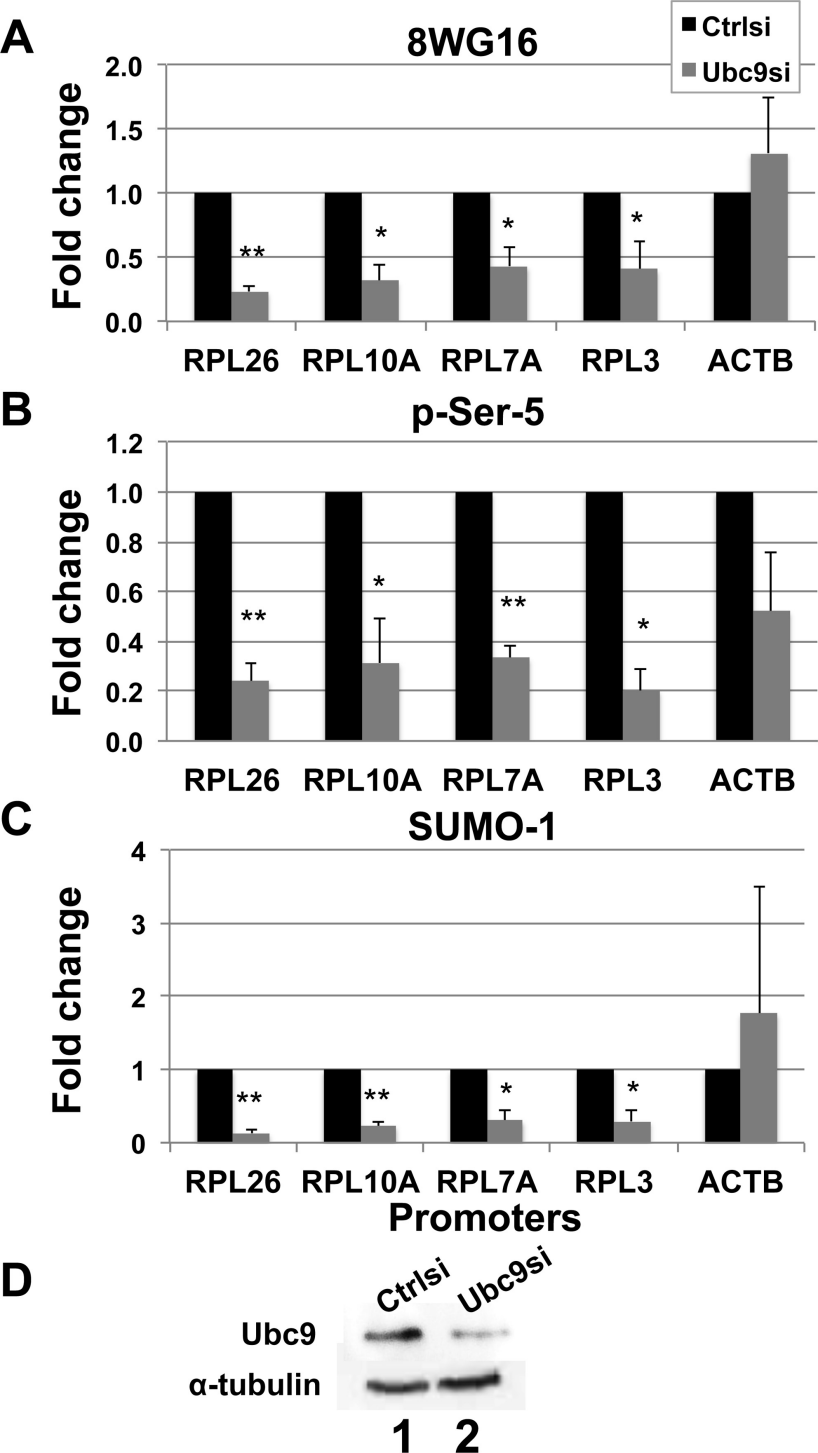
SUMOylation facilitates RNAPII recruitment on the active promoters. (**A-C**) Chromatin was isolated from control siRNA transfected cells (black) or UBC9 siRNA transfected cells (gray), and immunopurified using antibody specific for unphosphorylated RNAPII (8WG16, panel A), p-Ser-5 RNAPII (panel B) or SUMO-1 (panel C). Enrichment of the promoters of the indicated genes was detected by ChIP-qPCR, and to control for transfection efficiency, reactions were normalized to the control siRNA. Each column represents the mean ± SEM. *T*-test using the data from four biological replicates of ChIP–qPCR was conducted. The asterisk indicates statistical significance (*P ≤ 0.05; **P ≤ 0.01). Non-normalized results are presented in Supplementary Figure S1. (**D**) Immunoblots from the cells used in panels (A)–(C) were stained for UBC9 (*top*) and the loading control α-tubulin (*bottom*).

We have previously shown that SUMO-1 is enriched on the chromatin proteins bound to promoter regions of some of the most active genes during interphase, such as ribosomal protein (RP) encoding genes (8), which are highly abundant and constitutively transcribed by RNA polymerase II (RNAPII). Depletion of SUMO-1 caused a decrease in mRNA production, suggesting that the presence of SUMO-1 on the active promoter regions had a positive role for SUMOylation in transcriptional regulation (8). It had not been shown in human cells whether the decrease in mRNA abundance following depletion of SUMO-1 was due to a decrease in RNAPII binding. In yeast, which have only one SUMO isoform, SUMO protein is required for RNAPII recruitment on the constitutive genes during transcription initiation (9). We tested whether the SUMO-1 mark on the chromatin bound to a housekeeping promoter stimulates active RNAPII recruitment to promoters in mammalian cells. We investigated RNAPII occupancy under defective SUMOylation in HeLa cells by siRNA transfection targeting UBC9, the only SUMO-specific E2 ligase found in cells, and testing for RNAPII binding to promoter regions of ribosomal protein genes such as RPL3, RPL7A, RPL10A, RPL26, which were found enriched with SUMO-1 (8). In addition, we analyzed the β-actin promoter, which was not bound by SUMO-1, as a negative control. The enrichment of SUMO-1, RNAPII with phosphorylated serine-5 (pSer-5) of the carboxy-terminal domain (CTD) and RNAPII that is mainly unphosphorylated (8WG16) bound to promoters, was analyzed by ChIP-qPCR. The results were normalized to the control siRNA in each experiment to correct for a modest amount of variation in the percent of input obtained in each quantitative PCR assay. The results showed that upon UBC9 siRNA depletion, the occupancy of SUMO-1 on the promoters of RP genes significantly decreased 4- to 5-fold compared to controls (Figure 1C). We found that depletion of UBC9 and consequent defect in SUMOylation caused a decrease in the occupancy at these promoters of both unphosphorylated and phosphorylated form of RNAPII. Binding of the unphosphorylated RNAPII was decreased 2.5- to 5-fold. Binding of the pSer-5 form of RNAPII was decreased 4- to 5-fold compared to controls (Figure 1A and B). By contrast, the binding of either form of RNAPII to the β-actin promoter was not affected by depletion of UBC9 (Figure 1A and B). It is noteworthy that the effect on the pSer-5 form of RNAPII was of somewhat higher magnitude than the unphosphorylated form of RNAPII, suggesting that SUMOylation impacted the initiation process. The same results, shown in Supplementary Figure S1 but as individual experiments, clearly indicate the reduction in RNAPII and phospho-RNAPII associated with these promoters after depletion of the UBC9.

### SAFB is SUMO-1 modified and associated with RP gene promoters

Since SUMOylated chromatin-associated proteins and transcription factors are low in abundance, and SUMOylated targets are highly dynamic and rapidly reversed by SUMO proteases, a cell line called HeLa-SUMO1 that stably expresses SUMO-1 fused with a hexahistidine and biotinylated (HB) tag was used for isolation of SUMO-1-labeled chromatin proteins. SUMO-1 modified chromatin associated proteins were shown to be present during interphase and absent during mitosis (8). To identify the SUMO-1 substrates that mark the promoters during interphase, we purified the chromatin fraction from cells in either S phase or mitosis. The SUMOylated, and tagged, chromatin proteins were purified by metal ion affinity purification followed by avidin affinity purification with stringent washes. Purified proteins were then analyzed by mass spectrometry in order to identify proteins covalently bound to SUMO-1 during S phase and not during mitosis. Fifty-two proteins were identified by MS analysis, and the gene ontology (GO) term analysis showed that the top functions of those chromatin-associated proteins purified by virtue of binding to SUMO-1 in interphase were in RNA metabolism and protein synthesis pathways (Supplementary Table S1). For example, many RNA processing related factors, such as hnRNPs, PTB-associated splicing factor (SFPQ) and CPSF7, were detected as chromatin associated factors that were covalently SUMOylated. We suggest that SUMOylation of these splicing related factors on the promoters may serve as a link between transcriptional initiation and pre-mRNA splicing (Supplementary Table S1).

**Figure 2.**
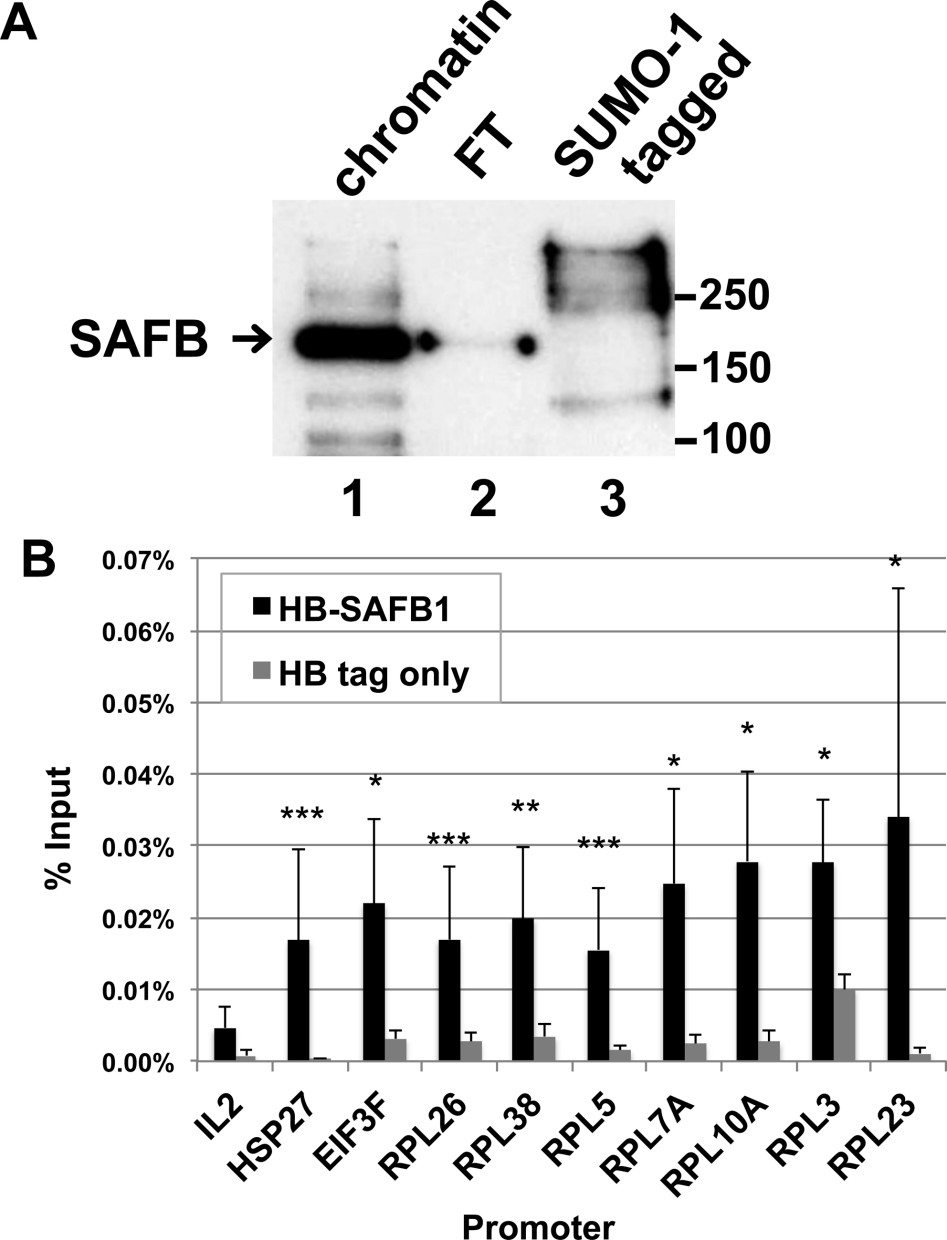
Identification of SAFB as SUMOylated substrate on the promoters. (**A**) Protein extracts from HeLa-SUMO-1 cells, which express his_6_-biotin-SUMO-1, were analyzed by immunoblots to track the SAFB through chromatin affinity purification with the chromatin fraction (lane 1), the protein unbound by the nickel-NTA matrix (FT; lane 2), and the eluate following metal ion affinity purification and biotin affinity purification (lane 3). (**B**) Verification of SAFB-1 enrichment on the SUMO-1 bound promoters. HeLa cells were transfected with SAFB-1 gene fused with the his_6_-biotin (HB) tag, and binding of the SAFB fusion protein to the indicated promoters was followed by ChIP-qPCR analysis. The results from three biological replicates are shown (mean ± SEM). The significance was evaluated by *T*-test, and the fold enrichment of samples was compared with results from cells transfected with an HB-tag only plasmid, which was used to control for nonspecific binding. The asterisk denotes the statistical significance (*P ≤ 0.05; **P ≤ 0.01; ***P ≤ 0.001).

The top 20 proteins identified by mass spectrometry are listed in order of highest score in Table 1. SAFB2 and SAFB1 were the proteins with the fifth highest score and the seventh highest score and had zero peptides detected in the mitotic chromatin but a high number of peptides in the S-phase chromatin. The peptide coverage for each protein is shown in Supplementary Figure S2. Interestingly, SAFB1/2 binding sites in promoter regions have been shown in another study (21), and two of the identified protein peaks representing SAFB binding sites in the pS2 and Hsp27 promoters (21) from that study coincided with genomic loci we observed to be bound by SUMO-1 in HeLa cells (data not shown). In another study, it had been found that SAFB was SUMOylated (26). We confirmed that SAFB was SUMOylated by affinity purification and immunoblot analysis, revealing a prominent SAFB protein in the input sample migrating at a position consistent with unmodified protein plus several bands of slower migration and lighter intensity. The unbound fraction contained a band of similar mass as the unmodified SAFB. By contrast, the bound, SUMO-1 tagged eluate contained multiple polypeptides that bound to SAFB specific antibody and that were shifted to slower migration (Figure 2A, lane 3), which we interpret to be consistent multiple SUMOylations of the two isoforms of the SAFB protein. In addition, we tested whether SAFB localized to the RP gene promoters by transfecting HeLa cells with SAFB-1 gene fused to the his_6_-biotin (HB) tag, and followed by ChIP-qPCR analysis. Transfection into control cells of a HB-tag only plasmid was used to control for nonspecific binding. SAFB-1 was found to associate with all eight of the RP promoters tested (Figure 2B).

**Table 1.** Mass spectrometry results of SUMOylated chromatin proteins

UniProt ID	Description	Spectra (M)	Spectra (S)	Max peptides	Seq tag coverage	Sequence coverage	Max score
P08670	Vimentin	34	103	33	67.00%	75.00%	1219
P02545	Prelamin-A/C	4	68	31	41.00%	47.00%	1146
Q15233	Non-POU domain-containing octamer-binding protein	12	78	28	44.00%	50.00%	950
P11387	DNA topoisomerase 1	37	26	20	21.00%	26.00%	545
Q14151	Scaffold attachment factor B2	–	34	17	17.00%	20.00%	500
P46060	Ran GTPase-activating protein 1	9	32	15	28.00%	34.00%	480
Q15424	Scaffold attachment factor B1	–	26	12	13.00%	16.00%	389
P23246	Splicing factor; proline- and glutamine-rich	3	23	12	20.00%	25.00%	343
O43143	Putative pre-mRNA-splicing factor ATP-dependent RNA helicase DHX15	2	26	11	12.00%	15.00%	328
P63165	Small ubiquitin-related modifier 1	14	34	7	42.00%	46.00%	307
P14866	Heterogeneous nuclear ribonucleoprotein L	2	20	11	24.00%	32.00%	268
Q8N684	Cleavage and polyadenylation specificity factor subunit 7	1	17	8	15.00%	19.00%	256
P07437	Tubulin beta chain	19	–	8	18.00%	23.00%	245
Q13263	Transcription intermediary factor 1-beta	–	20	11	13.00%	16.00%	239
P60709	Actin; cytoplasmic 1	15	5	6	16.00%	18.00%	198
Q00839	Heterogeneous nuclear ribonucleoprotein U	12	6	7	8.00%	11.00%	196
Q9Y2X3	Nucleolar protein 58	3	11	6	8.00%	13.00%	193
O75400	Pre-mRNA-processing factor 40 homolog A	–	9	4	4.00%	5.00%	176
P52272	Heterogeneous nuclear ribonucleoprotein M	13	–	6	9.00%	11.00%	152
Q71U36	Tubulin alpha-1A chain	12	1	5	15.00%	18.00%	149

### SAFB localizes SUMO-1 to promoters

**Figure 3.**
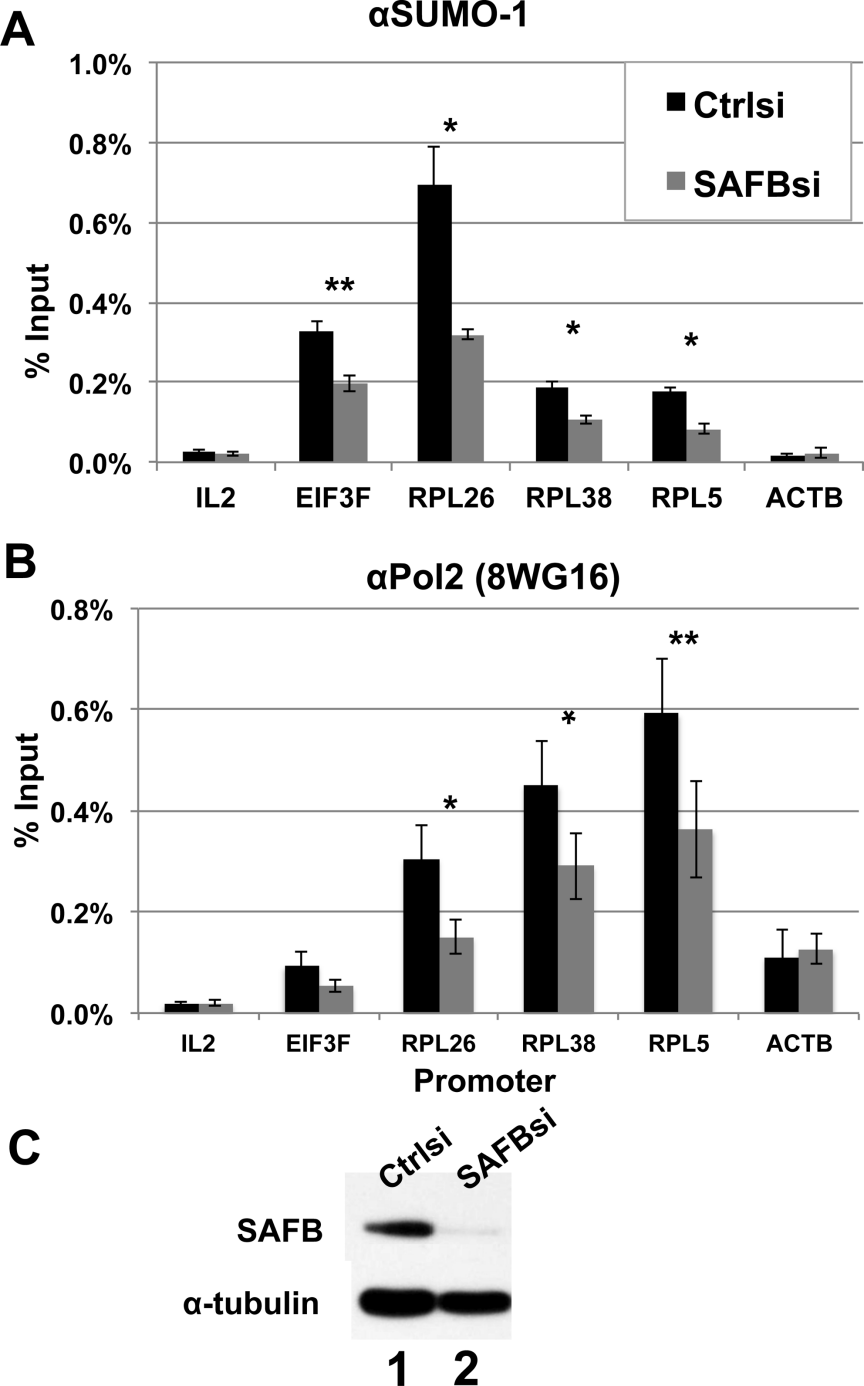
SAFB localizes SUMO-1 to promoters of RP genes. (**A**) Chromatin from HeLa cells was isolated from control siRNA transfected cells (black) or SAFB siRNA transfected cells (gray), and immunopurified with antibody specific to SUMO-1. Promoters of the indicated genes were detected by ChIP-qPCR. IL-2 was a negative control based on the gene expression and ChIP-seq data (8). *T*-test using the data from four biological replicates of ChIP-qPCR was conducted (*P-value ≤ 0.05; **P ≤ 0.01). (**B**) RNAPII binding on the promoters was detected by ChIP-qPCR as in panel (A). (**C**) Western blot analysis of SAFB or α-tubulin proteins was used to evaluate the depletion by the indicated siRNA transfection.

**Figure 4.**
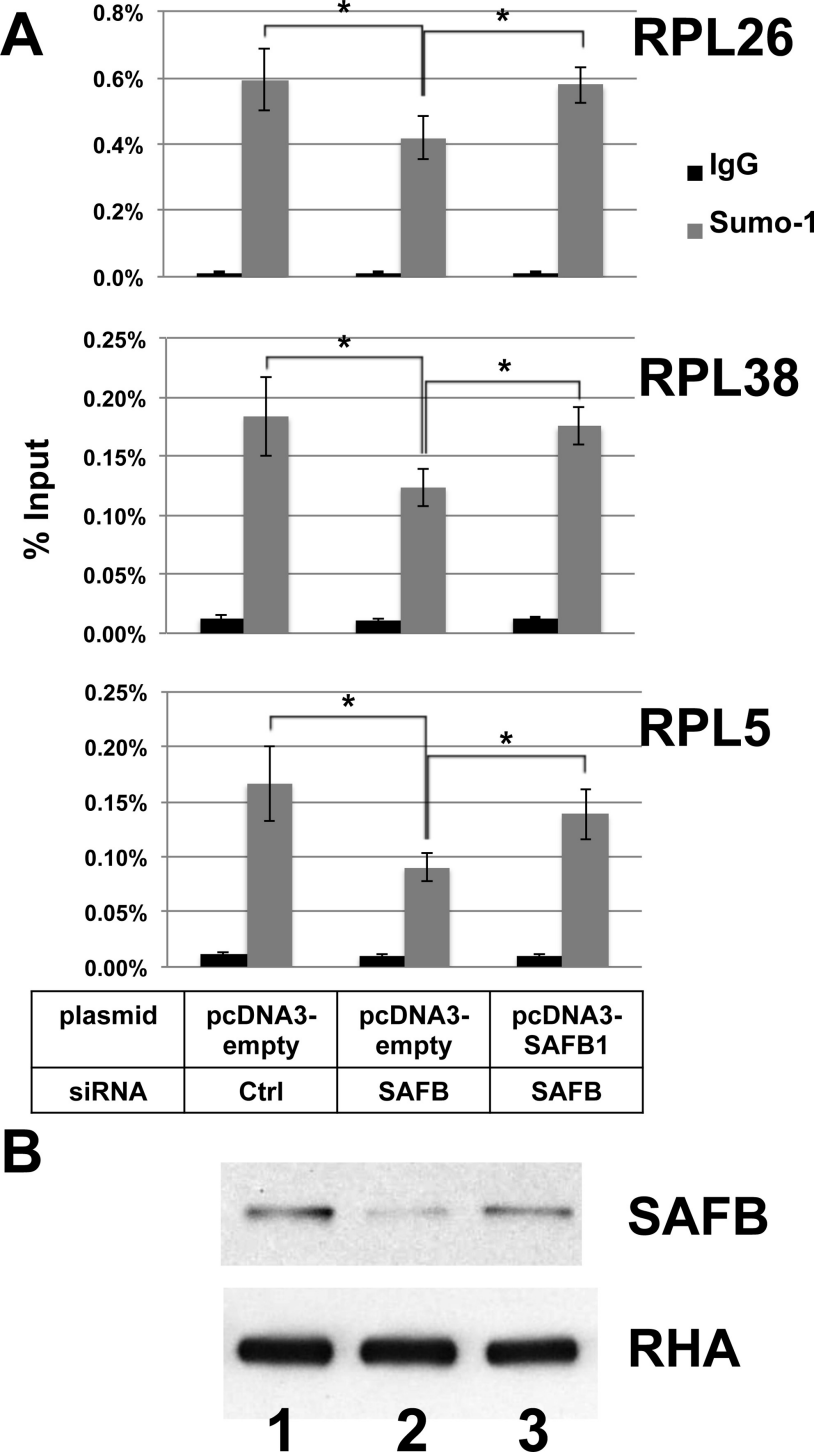
Expression of SAFB1 from a transfected plasmid rescued SUMOylation of promoter-bound chromatin. (**A**) A second set of siRNAs targeting the 3′-UTR of SAFB1 and the coding region of SAFB2 along with plasmid vector or plasmid for the expression of SAFB1 as indicated were transfected into HeLa cells. ChIP using control IgG (black) or SUMO-1 (gray) specific antibody was probed by qPCR for the promoters of the indicated genes. (**B**) Immunoblots for SAFB (*top*) or the unaffected loading control, RNA helicase A (RHA; *bottom*) were analyzed for control siRNA plus empty vector (lane 1), SAFB siRNAs plus empty vector (lane 2), and SAFB siRNAs plus SAFB1 expression plasmid (lane 3).

To determine if SAFB was responsible for the recruitment of SUMO-1 binding on the specific promoters, we tested whether depletion of SAFB affected the recruitment of SUMO-1 to promoters that we had previously characterized to be SUMO-1 bound (8). Since there are two highly related isoforms of SAFB, we depleted both homologs with siRNAs targeting SAFB1/2. Following siRNA transfection, immunoblot analysis showed that SAFB protein was depleted by >90% (Figure 3C). Consistent with earlier results, ChIP-qPCR analysis showed that under control conditions SUMO-1 and RNAPII were enriched on the RP gene promoter regions analyzed; IL2 was included as a negative control since it is not expressed in HeLa cells and its promoter had no detected SUMO-1. SAFB depletion caused a significant decrease in the SUMO-1 marks on the RP gene promoters, down to 40–50% compared to the controls (Figure 3A). Depletion of SAFB also caused a decrease in RNAPII occupancy on these promoters (Figure 3B). By contrast, when testing active genes that are not labeled by SUMO-1, such as β-actin, depletion of SAFB did not affect RNAPII occupancy on its promoter (Figure 3B), suggesting that SAFB facilitates RNAPII binding on the SUMO-1 labeled active genes. We further asked whether this phenomenon was caused by SAFB1. To this end, a second set of siRNAs for depletion of SAFB targeted the 3′UTR of SAFB1 and a second site in the ORF of SAFB2. Transfection of this second set of siRNAs also decreased ChIP specific for SUMO-1 at RP gene promoters, and expression of SAFB1 from a cotransfected plasmid rescued SUMO-1 binding to these promoters (Figure 4). These results clearly indicate that SAFB1 is a functionally relevant SUMO-1 target bound to the promoters, and these results support a model whereby the SUMOylation of SAFB stimulates RNAPII binding to target gene promoters.

### SAFB depletion caused down regulation of mRNA processing of RP genes

**Figure 5.**
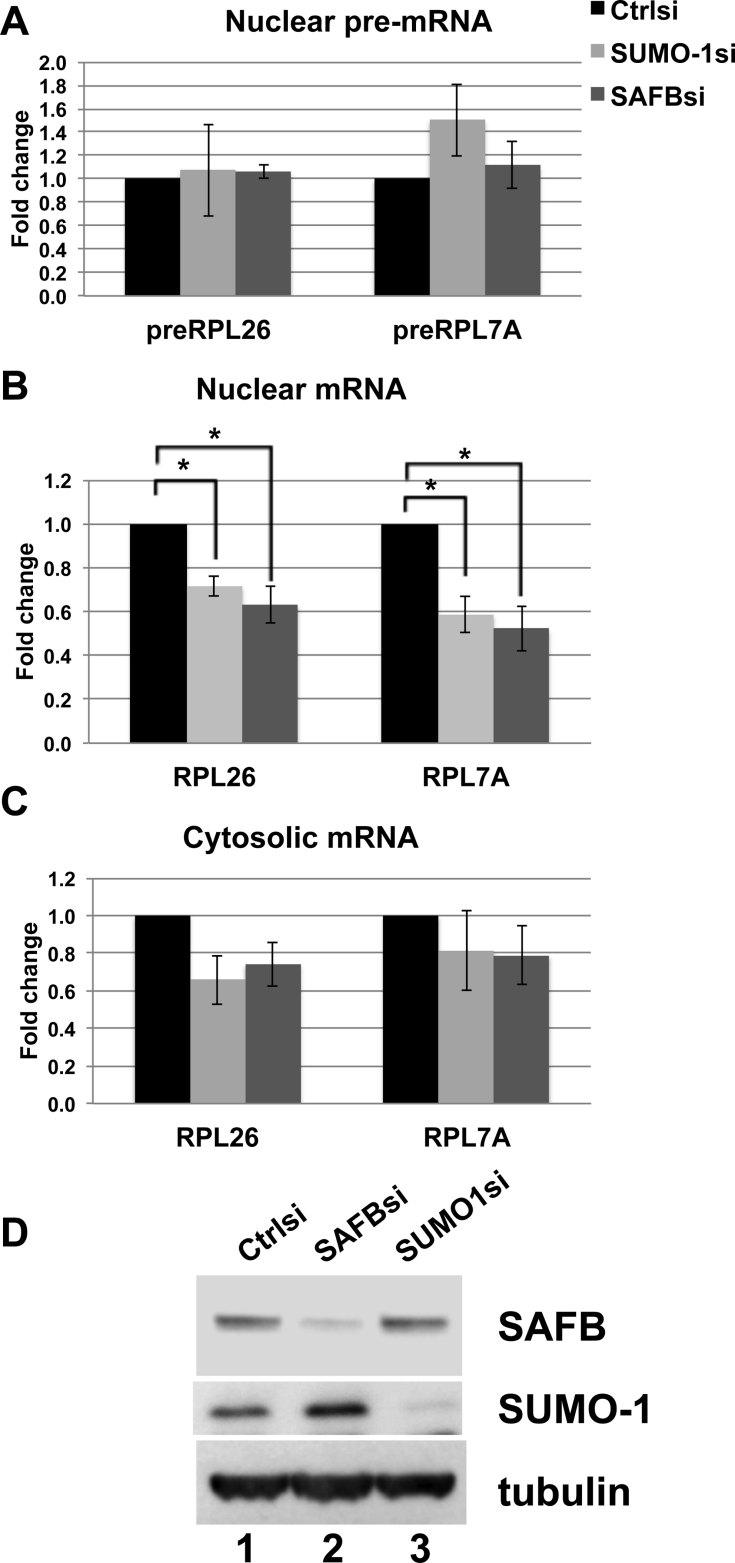
SAFB or SUMO-1 depletion caused down-regulation of nuclear spliced mRNA. RT-qPCR analysis of gene expression levels for the indicated genes 48 h after transfection using siRNAs specific for control, SUMO-1 or SAFB. (**A**) Abundance of intron-containing pre-mRNA from the nucleus was shown. (**B**) Spliced mRNA abundance in the nucleus is shown. (**C**) Spliced mRNA abundance in the cytoplasm is shown. The pre-mRNA/ mRNA expression level for each experiment was normalized to 18S rRNA as loading control (a non-SUMO-1-labeled gene) and normalized to the RNA levels detected in the sample from the control siRNA. Three biological replicates were done and error bars reflect the SEM. A *t*-test of equal expression between SUMO-1/SAFB and control siRNA using the data from three biological replications of RT-qPCR was conducted (*P-value ≤0 .05). (**D**) Immunoblots of SAFB, SUMO-1 or α-tubulin were used to assess the level of depletion of each protein.

**Figure 6.**
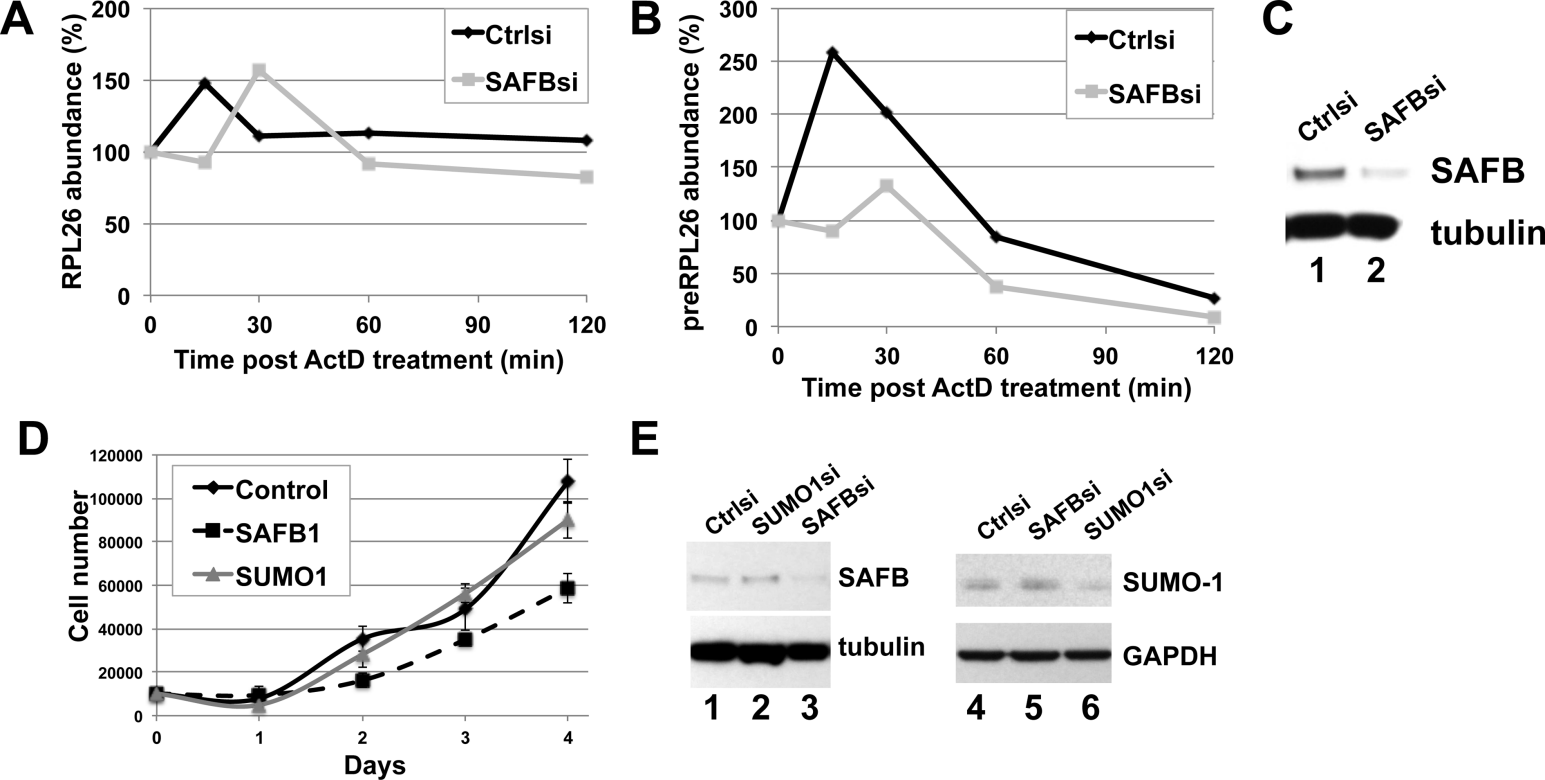
SAFB depletion does not affect the stability of the precursor or mature mRNA of RPL26 gene. (**A**–**C**) HeLa cells were transfected with SAFB or control siRNA for 48 h and then treated with either 2 μg/ml of actinomycin D (ActD) or DMSO as a control. The amount of RPL26 mRNA was monitored by qRT-PCR (panel A). Results show the concentrations of RNA species as the average of three independent replicates normalized to the concentration of the RNA in the DMSO vehicle sample. The abundance of intron-containing RPL26 pre-mRNA was monitored by qRT-PCR (panel B), and immunoblots from SAFB depletions were assessed (panel C). (**D**–**E**) The growth of HeLa cells following depletion of SAFB or SUMO-1 was assessed. Forty-eight hours post transfection with the siRNA, 10 000 cells were plated per well on day 0. Cell number was counted on days 1–4, as indicated. Results from three independent replicates are shown (panel D). *T*-test analysis was used to evaluate whether the growth rates were significantly different, and the p-value for comparing SUMO-1 depletion to the control depletion was 0.392 and for SAFB 0.153. Immunoblots taken at day 0 are shown (panel E).

We have shown previously that SUMO-1 marks the chromatin just upstream of the transcription start site of constitutive housekeeping genes and that the SUMO-1 mark stimulated transcription. In addition, the SUMO-1 mark was also found enriched on exons in the human genome, suggesting a potential role for facilitating splicing (8). Given that SAFB interacts with the CTD of RNAPII (13), that SAFB1 is involved in recruitment of SUMO-1 and RNAPII on the promoters (Figures 3 and 4), and that our mass spectrometry results indicated multiple SUMOylated splicing factors (Supplementary Table S1), we tested whether SAFB depletion may affect mRNA expression of the RP genes at the level of pre-mRNA splicing. We investigated the RNA processing of two RP genes, RPL26 and RPL7a, by quantifying RNA containing the exon-exon junction for spliced mRNA in the nucleus, and we quantified the abundance of the intron-exon junctions for measuring pre-mRNA concentration. We are confident that the PCR product from the unspliced pre-mRNA did not result from contamination of genomic DNA since the samples were thoroughly treated with DNase and since the pre-mRNA decreased over time following actinomycin D treatment (Figure 6), and such a decrease over time is inconsistent with genomic DNA contamination. The RT-qPCR analysis showed that depletion of either SUMO-1 or SAFB did not affect the abundance of the primary transcripts relative to the control in the nucleus (Figure 5A), but the spliced mRNA purified from the nucleus was less abundant in SUMO-1 or SAFB depleted cells. This result suggested that SUMO-1 and SAFB are involved in mRNA processing (Figure 5B). It was surprising that the decrease in splicing did not result in an excess accumulation of nuclear pre-mRNA. We suggest that the decrease in initiation (Figure 1) was balanced by the decrease in RNA processing (Figure 5B) to yield little change to the unspliced pre-mRNA (Figure 5A). We also tested the mature mRNA in the cytosol, and the RP genes were reduced due to either SUMO-1 or SAFB depletion (Figure 5C).

To further investigate the effect of SAFB on RNA metabolism, we asked whether depletion of SAFB affected mRNA stability. Actinomycin D was included in media to block new mRNA transcription, and we harvested RNA species at different time points. We found that there is no change of mature mRNA, suggesting the RP RNA is fairly stable for at least 2 h, independent of SUMOylation or of SAFB (Figure 6A). The precursor mRNA decreased at a similar rate from cells that were SAFB depleted or control siRNA transfected, and the difference in splicing rate for SAFB depleted is not detected when in the presence of actinomycin D, indicating that RNA stability is not affected by SAFB depletion (Figure 6B). It was noted that following addition of actinomycin D to the medium, there was a rapid and reproducible spike in the abundance of the RPL26 pre-spliced RNA and spliced RNA. This spike was observed in all three replicates of the control depleted cells as well as the SAFB depleted cells, though the timing of the spike was perhaps delayed in the SAFB depleted cells. Though the cause of this spike in RNA concentration was not clear, its presence did not affect the interpretation that the RNA stability was not affected by SAFB depletion. Taken together with the other experiments, the newly transcribed mRNA in the nucleus was down regulated in SAFB depleted cells (Figure 4B), and we suggest that SUMOylated SAFB stimulates mRNA splicing, and the down regulation of mRNA in the nucleus was not due to the RNA stability.

Since ribosomal protein expression is highly sensitive to cell proliferation rates, we tested whether depletion of SAFB or SUMO-1 affected cell proliferation. We depleted SAFB and SUMO-1 from HeLa cells as usual (Figure 6E), and seeded 10 000 cells per well and measured the cell number each day for 4 days. The proliferation rates were not statistically different (Figure 6D), indicating that the changes in RP RNA abundance observed in Figure 5 were not secondary to significant changes in growth rates.

## DISCUSSION

In this study, we discovered that SUMO-1 binds to the chromatin at promoters of ribosomal protein genes via the scaffold attachment factor, SAFB. We found that SAFB is SUMOylated, and depletion of SAFB caused a decrease of SUMO-1 association with the promoters on the chromatin. These promoters, encoding RP genes and translation factors, are among the most active RNAPII promoters in the cell, and SUMO-1-tagged SAFB stimulates both the recruitment of RNAPII and the splicing of the product RNAs. In addition, a number of RNA processing factors were found to be SUMO-1 targets, suggesting that SUMO-1 marks on the promoter during the transcription cycle can be important for determining the efficiency of pre-mRNA splicing. The SUMO-1-SAFB axis revealed in this study, defines a fast track for gene expression; blocking this pathway via RNAi mediated depletion did not cause a total inhibition of transcription and processing but rather a decrease toward the expression levels of most protein-encoding genes (8).

The SAFB1 protein has been reported to be covalently modified by SUMO-1 as well as SUMO-2/3, and this modification is associated with transcriptional repression activity (26). The lysine acceptors for the SAFB1-mediated co-repressor function were identified to be K231 and K294, and these modifications were important for the SAFB1 recruitment of HDAC3 to these promoters and transcriptional repression (26). In the current study, only SUMO-1 was tested, and it remains to be tested if SUMO-2/3 modification has similar effects on stimulating transcriptional initiation and RNA processing. In addition, the SUMO E3 ligase PIAS1 and the SENP1 SUMO protease were found to regulate the conjugation and removal of SUMO proteins on to SAFB1 (26). It will be of great interest to determine whether these enzymes similarly affect the ribosomal protein transcription and RNA process characterized in the current study. It was striking that SUMOylation of SAFB1 was associated with transcriptional repression of estrogen receptor regulated genes (21,26), but in the current study this modification resulted in transcriptional stimulation of RP genes. It will be of great interest to characterize the gene-specific activities of the SUMO1 modification of SAFB.

SUMOylation of multiple proteins in a single pathway that stimulate the DNA repair process has been described (27), and we suggest that a similar synergy exists for SUMOylation of SAFB and splicing factors for coupling transcription initiation with processing. The concept of linking transcription initiation with splicing has been described; a previous study showed that the strength of a promoter-bound activator could also affect the efficiency of constitutive splicing and 3′-end cleavage of different reporter pre-mRNAs (28). This activator-dependent increase in pre-mRNA processing efficiency required the RNAPII CTD (29). In this study, we found that the link between initiation and mRNA processing of RP genes depended on the SUMOylation of SAFB.

Nuclear function depends on organizing platforms for establishing structural and functional domains in the nucleus. In this study, we found that SUMOylation facilitates RNAPII recruitment on the constitutive promoters, which is consistent with the observation in yeast (9). In addition, SAFB depletion caused down-regulation of SUMO-1 and RNAPII binding on those highly active promoters, and the mature mRNA expression, suggesting that SAFB links transcription initiation and splicing for the most active RNAPII transcripts in the cell. There are emerging studies showing the importance of SAFB on regulation of chromatin architecture. For example, SAFB1 participates in chromatin remodeling by interacting with ATP-dependent chromatin modifying proteins such as CHD1 (18). The CHD1 protein binds to a histone mark in promoters and regulates early transcription events including splicing (19). It is possible that SAFB interaction with CHD1 is in part regulated by SUMO-1 and is interacting with the CHD1-spliceosome complex. A recent study reported that SAFB1 regulates chromatin accessibility in response to genotoxic stress, and it is transiently recruited to DNA damage sites for efficient signaling and the downstream phosphorylation of chromatin (23). Another study showed that SAFB1 is associated with the activation of skeletal muscle gene expression during myogenic differentiation by facilitating the transition of promoter sequences from a repressive chromatin structure to one that is transcriptionally active (22). It is well-established that the localization of the splicing SR proteins coincides with the sites of active RNAPII during transcription (29). Considering that both SAFB and splicing SR proteins interact with the RNAPII CTD, and the concept of a transcriptosome complex has been suggested to link transcription initiation and splicing (13), we suggest that the SUMOylation of SAFB participates in this process. Interestingly, SAFB interacts with SF2/ASF *in vivo* (13), and it has been reported recently that SF2/ASF functions as a cofactor to enhance SUMOylation through the E3 ligase PIAS1 (30). Therefore, it is possible that SAFB and SF2/ASF may serve as the functional link between the RNA processing and SUMOylation machinery.

Taken together, the results of this study suggest a novel function for SAFB in regulation of gene expression by coordinating transcriptional initiation and RNA processing. It is possible that SUMOylated SAFB associates with the CTD of the initiating RNAPII and travels with RNAPII as it synthesizes nascent pre-mRNA, and subsequently facilitates the assembly of splicing complexes and splicing on the first intron to emerge, and further coordinates transcription and pre-mRNA processing levels.

## SUPPLEMENTARY DATA

Supplementary Data are available at NAR Online.

SUPPLEMENTARY DATA

## References

[1] Nacerddine K., Lehembre F., Bhaumik M., Artus J., Cohen-Tannoudji M., Babinet C., Pandolfi P.P., Dejean A. (2005). The SUMO pathway is essential for nuclear integrity and chromosome segregation in mice. Dev. Cell.

[2] Geiss-Friedlander R., Melchior F. (2007). Concepts in sumoylation: a decade on. Nat. Rev. Mol. Cell Biol..

[3] Ouyang J., Shi Y., Valin A., Xuan Y., Gill G. (2009). Direct binding of CoREST1 to SUMO-2/3 contributes to gene-specific repression by the LSD1/CoREST1/HDAC complex. Mol. Cell.

[4] Shiio Y., Eisenman R.N. (2003). Histone sumoylation is associated with transcriptional repression. Proc. Natl. Acad. Sci. U.S.A..

[5] Guo B., Sharrocks A.D. (2009). Extracellular signal-regulated kinase mitogen-activated protein kinase signaling initiates a dynamic interplay between sumoylation and ubiquitination to regulate the activity of the transcriptional activator PEA3. Mol. Cell. Biol..

[6] Lyst M.J., Nan X., Stancheva I. (2006). Regulation of MBD1-mediated transcriptional repression by SUMO and PIAS proteins. EMBO J..

[7] Rodriguez M.S., Desterro J.M., Lain S., Midgley C.A., Lane D.P., Hay R.T. (1999). SUMO-1 modification activates the transcriptional response of p53. EMBO J..

[8] Liu H.W., Zhang J., Heine G.F., Arora M., Gulcin Ozer H., Onti-Srinivasan R., Huang K., Parvin J.D. (2012). Chromatin modification by SUMO-1 stimulates the promoters of translation machinery genes. Nucleic Acids Res..

[9] Rosonina E., Duncan S.M., Manley J.L. (2010). SUMO functions in constitutive transcription and during activation of inducible genes in yeast. Genes Dev..

[10] Neyret-Kahn H., Benhamed M., Ye T., Le Gras S., Cossec J.C., Lapaquette P., Bischof O., Ouspenskaia M., Dasso M., Seeler J. (2013). Sumoylation at chromatin governs coordinated repression of a transcriptional program essential for cell growth and proliferation. Genome Res..

[11] Oesterreich S., Lee A.V., Sullivan T.M., Samuel S.K., Davie J.R., Fuqua S.A. (1997). Novel nuclear matrix protein HET binds to and influences activity of the HSP27 promoter in human breast cancer cells. J. Cell. Biochem..

[12] Arao Y., Kuriyama R., Kayama F., Kato S. (2000). A nuclear matrix-associated factor, SAF-B, interacts with specific isoforms of AUF1/hnRNP D. Arch. Biochem. Biophys..

[13] Nayler O., Stratling W., Bourquin J.P., Stagljar I., Lindemann L., Jasper H., Hartmann A.M., Fackelmayer F.O., Ullrich A., Stamm S. (1998). SAF-B protein couples transcription and pre-mRNA splicing to SAR/MAR elements. Nucleic Acids Res..

[14] Melnik S., Deng B., Papantonis A., Baboo S., Carr I.M., Cook P.R. (2011). The proteomes of transcription factories containing RNA polymerases I, II or III. Nat. Methods.

[15] Chiodi I., Biggiogera M., Denegri M., Corioni M., Weighardt F., Cobianchi F., Riva S., Biamonti G. (2000). Structure and dynamics of hnRNP-labelled nuclear bodies induced by stress treatments. J. Cell Sci..

[16] Forrester W.C., van Genderen C., Jenuwein T., Grosschedl R. (1994). Dependence of enhancer-mediated transcription of the immunoglobulin mu gene on nuclear matrix attachment regions. Science.

[17] Jenke A.C., Stehle I.M., Herrmann F., Eisenberger T., Baiker A., Bode J., Fackelmayer F.O., Lipps H.J. (2004). Nuclear scaffold/matrix attached region modules linked to a transcription unit are sufficient for replication and maintenance of a mammalian episome. Proc. Natl. Acad. Sci. U.S.A..

[18] Tai H.H., Geisterfer M., Bell J.C., Moniwa M., Davie J.R., Boucher L., McBurney M.W. (2003). CHD1 associates with NCoR and histone deacetylase as well as with RNA splicing proteins. Biochem. Biophys. Res. Commun..

[19] Sims R.J. 3rd, Millhouse S., Chen C.F., Lewis B.A., Erdjument-Bromage H., Tempst P., Manley J.L., Reinberg D. (2007). Recognition of trimethylated histone H3 lysine 4 facilitates the recruitment of transcription postinitiation factors and pre-mRNA splicing. Mol. Cell.

[20] Oesterreich S., Zhang Q., Hopp T., Fuqua S.A., Michaelis M., Zhao H.H., Davie J.R., Osborne C.K., Lee A.V. (2000). Tamoxifen-bound estrogen receptor (ER) strongly interacts with the nuclear matrix protein HET/SAF-B, a novel inhibitor of ER-mediated transactivation. Mol. Endocrinol..

[21] Hammerich-Hille S., Kaipparettu B.A., Tsimelzon A., Creighton C.J., Jiang S., Polo J.M., Melnick A., Meyer R., Oesterreich S. (2010). SAFB1 mediates repression of immune regulators and apoptotic genes in breast cancer cells. J. Biol. Chem..

[22] Hernandez-Hernandez J.M., Mallappa C., Nasipak B.T., Oesterreich S., Imbalzano A.N. (2013). The Scaffold attachment factor b1 (Safb1) regulates myogenic differentiation by facilitating the transition of myogenic gene chromatin from a repressed to an activated state. Nucleic Acids Res..

[23] Altmeyer M., Toledo L., Gudjonsson T., Grofte M., Rask M.B., Lukas C., Akimov V., Blagoev B., Bartek J., Lukas J. (2013). The chromatin scaffold protein SAFB1 renders chromatin permissive for DNA damage signaling. Mol. Cell.

[24] Xu H., Yang L., Freitas M.A. (2008). A robust linear regression based algorithm for automated evaluation of peptide identifications from shotgun proteomics by use of reversed-phase liquid chromatography retention time. BMC Bioinformatics.

[25] Thompson N.E., Burgess R.R. (1996). Immunoaffinity purification of RNA polymerase II and transcription factors using polyol-responsive monoclonal antibodies. Methods Enzymol..

[26] Garee J.P., Meyer R., Oesterreich S. (2011). Co-repressor activity of scaffold attachment factor B1 requires sumoylation. Biochem. Biophys. Res. Commun..

[27] Psakhye I., Jentsch S. (2012). Protein group modification and synergy in the SUMO pathway as exemplified in DNA repair. Cell.

[28] Rosonina E., Bakowski M.A., McCracken S., Blencowe B.J. (2003). Transcriptional activators control splicing and 3′-end cleavage levels. J. Biol. Chem..

[29] Mortillaro M.J., Blencowe B.J., Wei X., Nakayasu H., Du L., Warren S.L., Sharp P.A., Berezney R. (1996). A hyperphosphorylated form of the large subunit of RNA polymerase II is associated with splicing complexes and the nuclear matrix. Proc. Natl. Acad. Sci. U.S.A..

[30] Pelisch F., Gerez J., Druker J., Schor I.E., Munoz M.J., Risso G., Petrillo E., Westman B.J., Lamond A.I., Arzt E. (2010). The serine/arginine-rich protein SF2/ASF regulates protein sumoylation. Proc. Natl. Acd. Sci. U.S.A..

