# Supplementary material for: The chromatin scaffold protein SAFB1 localizes SUMO-1 to the promoters of ribosomal protein genes to facilitate transcription initiation and splicing
Source: Nucleic Acids Res. 2015 Mar 23;43(7):3605–13. doi: 10.1093/nar/gkv246 (PMC4402547; doi:10.1093/nar/gkv246)
Supplement: SUPPLEMENTARY DATA [file supp_43_7_3605__index.html]

The chromatin scaffold protein SAFB1 localizes SUMO-1 to the promoters of ribosomal protein genes to facilitate transcription initiation and splicing — The chromatin scaffold protein SAFB1 localizes SUMO-1 to the promoters of ribosomal protein genes to facilitate transcription initiation and splicing — SUPPLEMENTARY DATA 

# The chromatin scaffold protein SAFB1 localizes SUMO-1 to the promoters of ribosomal protein genes to facilitate transcription initiation and splicing

## SUPPLEMENTARY DATA

**Files in this Data Supplement:**

- SUPPLEMENTARY DATA
