# Supplementary material for: The chromatin scaffold protein SAFB1 localizes SUMO-1 to the promoters of ribosomal protein genes to facilitate transcription initiation and splicing
Source: Nucleic Acids Res. 2015 Mar 23;43(7):3605–13. doi: 10.1093/nar/gkv246 (PMC4402547; doi:10.1093/nar/gkv246)
Supplement: SUPPLEMENTARY DATA [file supp_gkv246_nar-03226-x-2014-File008.pdf]

Supplementary information

The chromatin scaffold protein SAFB1 localizes SUMO-1 to the promoters of ribosomal protein genes to facilitate transcription initiation and splicing.

Hui-wen Liu[1], Tapahsama Banerjee[1], Xiaoyan Guan[2], Michael A. Freitas[2], and Jeffrey D. Parvin[1,*].

[1]Department of Biomedical Informatics,

[2]Department of Molecular Virology, Immunology, and Medical Genetics,

Comprehensive Cancer Center, The Ohio State University, Columbus, OH 43210, USA

To whom correspondence should be addressed. Tel: +1-614-292-0523; Fax: +1-614-688-6600; Email: Jeffrey.Parvin@osumc.edu

Present address: Hui-wen Liu, Oregon Health and Science University, Portland, OR 97239, USA; Xiaoyan Guan (Florida State University, Tallahassee, FL)

Legends for Supplementary Figures

Supplementary Figure S1. SUMOylation facilitates RNAPII recruitment on the active promoters.

Results from Figure 1 are shown as individual experiments; in each case the purification of a promoter DNA fragment is given as the ratio of the indicated antibody to the IgG control antibody. The immunoprecipitating antibody was specific for unphosphorylated RNAPII (8WG16; top row), RNAPII with phosphorylated Ser5 on the carboxy-terminal domain (middle row), and SUMO-1 (bottom row).

Supplementary Figure S2. Mass spectrometry results of SUMOylated chromatin proteins in interphase.

Mass spectrometry results are shown for SAFB2 (panel A) and SAFB1 (panel B). Peptides detected are in colored fonts; if the amino acid residue was sequenced in two directions, it is indicated in red font.

Supplementary Table S1 Gene ontology (GO) analysis of the SUMO-1 enriched chromatin binding proteins during S phase in HeLa cells.

Name	*p*-value	# Molecules
RNA post-transcriptional modification	1.92E-09 – 6.25E-03	FBL, HNRNPA0, HNRNPA1, HNRNPC, HNRNPL, HNRNPU, NOP58, SAFB, SF3A2, SFPQ, SNRNP200
DNA replication, Recombination and Repair	1.39E-07 – 4.12E-02	HNRNPA1, HNRNPC, HNRNPU, CTCF, SAFB, FBL, KRT7, LMNA, MCM3AP, TOP1
Post-translational modification	7.66E-06 – 3.88E-02	SUMO1, SUMO2, TRIM28
Gene expression	1.95E-05 – 3.88E-02	CCNT1, CTCF, DHX15, GTF2I, HNRNPA1, HNNPC, KRT7, LMNA, MATR3, MCM3AP, SAFB, SFPQ, SUMO1, SUMO2, TFAP2A, TOP1, TRIM28, ZNF281, ZNF384
Cell morphology	3.56E-05 – 4.36E-02	FBL, HNRNPA0, HNRNPA1, HNRNPC, HNRNPL, HNRNPU, NOP58, SAFB, SF3A2, SFPQ, SNRNP200

Supplementary Table S2 Primers and siRNAs used in this research

Primers for RT-qPCR

Gene name (Refseq)	Sequence (5' to 3')
RPL23	GGTGGGCGGGGCGTTAAAGT
	CCCACCACGTCCTCGCTTCG
RPL26	AGCGGGAGCGGCCAAAATGA
	TTCCCGCTGCACCCGTTCAA
Pre-RPL26 (exon1/intron1)	GGCTTTCCGTTCGAGGATCT
	TTAGGCATCCACCTACCCCA
Pre-RPL7A (exon4/intron4)	GACGTCCCAACGAAGAGACC
	CCCCCAGTGTTCACCCTAAG
18s rRNA	TTCGGAACTGAGGCCATGAT
	TTTCGCTCTGGTCCGTCTTG

Primers for ChIP-qPCR

Gene name (Refseq)	Sequence (5' to 3')
IL2	TCTGCCTGCTTTCTGTGAAACTCAA
	GGACAAGCCTCATCCCAAACTCCA
EIF3F	GTTTCTCTTCGAACGCCGT
	GAGCACTGAAATAGTCCCGC
RPS27	CAGGATTTCCGCTTTCGCTC
	ACAGAACAGCGAGATCTCCG
RPL26	GACCTATGTCTCTCGGAGCG
	GTTTTGCAATCCCTCGCAGT
RPL38	TGATCCTCGGCAGGCACCGT
	CAGCAAGCAGCAACCGGGGA
RPL5	CGTCACTGGCGTGACCGTCC
	CTGCGGAACAGAGACCGGCG
RPL7A	CCGCCGCCCAAGATGGTGAG
	GGCAGCGGATACAGCCGGAA
RPL10A	GGCGCTCAGGACTGCGACAA
	GCTCCTCGGTCCTACCCGCA
RPL3	CCTTCGGAGTGCACCAGCGG
	TTGCTTTAGGGGCACGGGCG
RPL23	AATCCGCCAGCCACTGCACG
	TTGCTCCGGCCACGTGAGGA
HSP27	GTTCCAGATGAGGGCTGAAC
	TCTGGACGTCTGCTCAGAAA

siRNAs

Gene name	Sequence
Ubc9	CAAAAAAUCCCGAUGGCAC
SUMO-1	CUGGGAAUGGAGGAAGAAG
SAFB1	GUAAUCCUGACGAAAUUGA
SAFB1-3'UTR	AAGGCUAUGUUCUGUUAGGAG
SAFB2	GAAUAGCAGUGCUCCAGAU
SAFB2-ORF	GAAAUUGGCAUCGAGUUAGAA

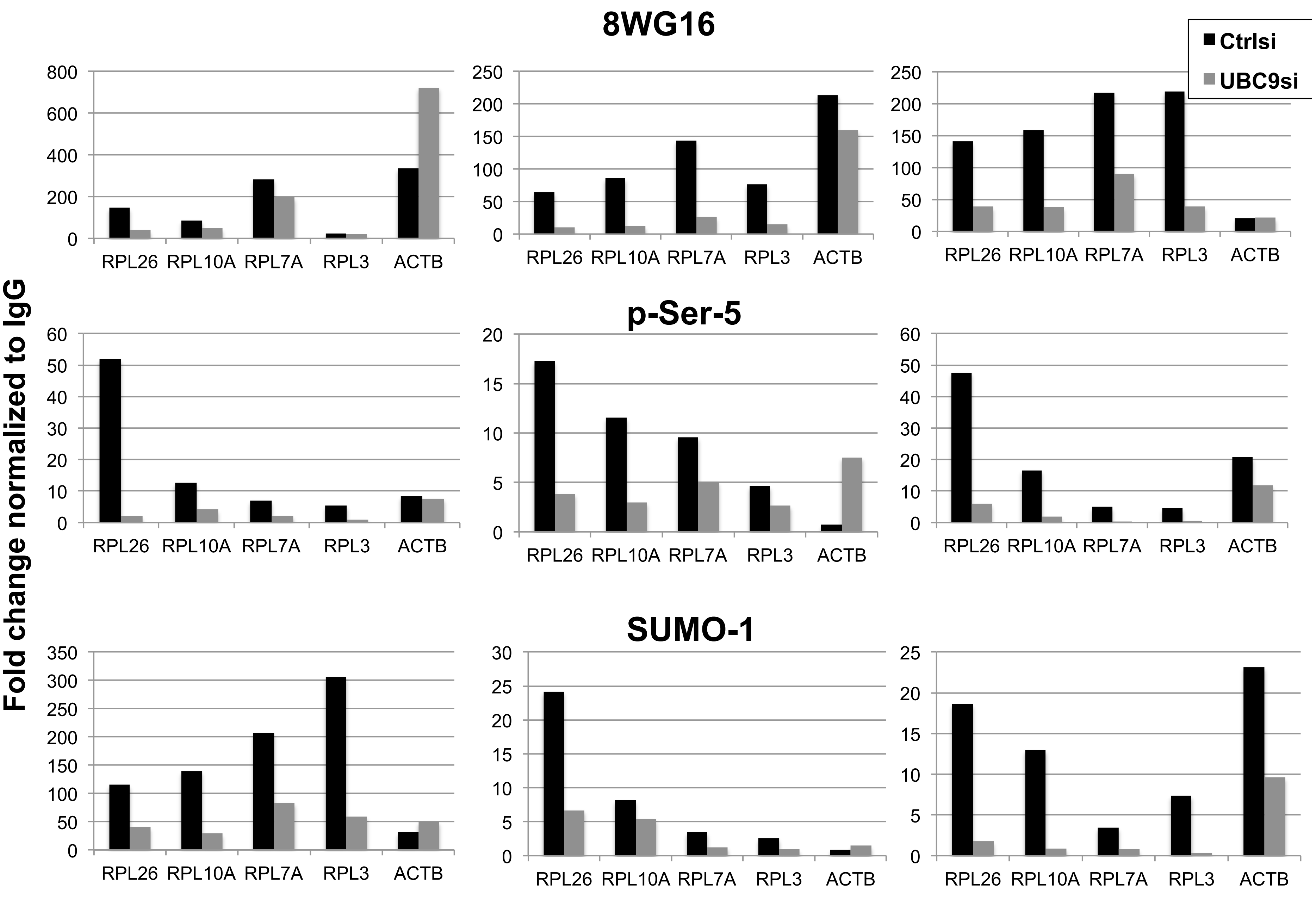

Supplementary Figure S1

Supplementary Figure S2

A.

HIT 7

Protein Mass: 107408.078 (monoisotopic) 107472.709(average)   Protein Score: **500**   Protein pp: **7999.2**
sp|Q14151|SAFB2_HUMAN Scaffold attachment factor B2 OS=Homo sapiens GN=SAFB2 PE=1 SV=1

Sequence:

```
 001 MAETLPGSGD SGPGTASLGP GVAETGTRRL SELRVIDLRA ELKKRNLDTG GNKSVLMERL KKAVKEEGQD PDEIGIELEA TSKKSAKRCV KGLKMEEEGT  100
 101 EDNGLEDDSR DGQEDMEASL ENLQNMGMMD MSVLDETEVA NSSAPDFGED GTDGLLDSFC DSKEYVAAQL RQLPAQPPEH AVDGEGFKNT LETSSLNFKV  200
 201 TPDIEESLLE PENEKILDIL GETCKSEPVK EESSELEQPF AQDTSSVGPD RKLAEEEDLF DSAHPEEGDL DLASESTAHA QSSKADSLLA VVKREPAEQP  300
 301 GDGERTDCEP VGLEPAVEQS SAASELAEAS SEELAEAPTE APSPEARDSK EDGRKFDFDA CNEVPPAPKE SSTSEGADQK MSSFKEEKDI KPIIKDEKGR  400
 401 VGSGSGRNLW VSGLSSTTRA TDLKNLFSKY GKVVGAKVVT NARSPGARCY GFVTMSTSDE ATKCISHLHR TELHGRMISV EKAKNEPAGK KLSDRKECEV  500
 501 KKEKLSSVDR HHSVEIKIEK TVIKKEEKIE KKEEKKPEDI KKEEKDQDEL KPGPTNRSRV TKSGSRGMER TVVMDKSKGE PVISVKTTSR SKERSSKSQD  600
 601 RKSESKEKRD ILSFDKIKEQ RERERQRQRE REIRETERRR EREQREREQR LEAFHERKEK ARLQRERLQL ECQRQRLERE RMERERLERE RMRVERERRK  700
 701 EQERIHRERE ELRRQQEQLR YEQERRPGRR PYDLDRRDDA YWPEGKRVAM EDRYRADFPR PDHRFHDFDH RDRGQYQDHA IDRREGSRPM MGDHRDGQHY  800
 801 GDDRHGHGGP PERHGRDSRD GWGGYGSDKR LSEGRGLPPP PRGGRDWGEH NQRLEEHQAR AWQGAMDAGA ASREHARWQG GERGLSGPSG PGHMASRGGV  900
 901 AGRGGFAQGG HSQGHVVPGG GLEGGGVASQ DRGSRVPHPH PHPPPYPHFT RRY                                                   953
```

Sequence Coverage: **20%**
Sequence Tag Coverage: **17%**

Index	scan#	charge	score	pp	pp_2	pp_{tag}	m/z	MW(obs)	MW	delta	miss	Unique	sequence + modifications [start:end]
2294	1715	+3	49	10.1	9.5	9.6	753.3702	2258.0962	2258.1034	-0.0073	1	√	AVKEEGQDPDEIGIELEATSK
2295	1718	+3	29	6.4	6.1	8.8	753.3718	2258.1007	2258.1034	-0.0027	1	√	AVKEEGQDPDEIGIELEATSK [63:83]
2402	1598	+4	38	9.4	5.7	3.4	597.3022	2386.1869	2386.1984	-0.0115	2	√	AVKEEGQDPDEIGIELEATSKK
2403	1599	+3	65	19.9	11.8	13.4	796.0690	2386.1925	2386.1984	-0.0059	2	√	AVKEEGQDPDEIGIELEATSKK
2404	1607	+3	47	14.8	8.6	14.3	796.0735	2386.2059	2386.1984	0.0075	2	√	AVKEEGQDPDEIGIELEATSKK [63:84]
308	887	+2	28	9.5	13.3	4.8	475.2582	949.5091	949.5102	-0.0011	0	√	EYVAAQLR [164:171]
2829	2030	+3	18	8.1	3.0	4.3	1018.8458	3054.5229	3054.5167	0.0062	1	√	QLPAQPPEHAVDGEGFKNTLETSSLNFK
2830	2038	+3	15	5.4	2.9	1.4	1018.8477	3054.5286	3054.5167	0.0119	1	√	QLPAQPPEHAVDGEGFKNTLETSSLNFK [172:199]
2728	1592	+3	65	11.2	11.8	9.9	950.0995	2848.2841	2848.3119	-0.0278	1	×	SEPVKEESSELEQPFAQDTSSVGPDR [226:251]
3040	1874	+4	101	17.0	11.9	12.0	889.9098	3556.6173	3556.6198	-0.0025	1	×	KLAEEEDLFDSAHPEEGDLDLASESTAHAQSSK
3041	1883	+3	138	28.9	19.7	23.9	1186.2136	3556.6263	3556.6198	0.0065	1	×	KLAEEEDLFDSAHPEEGDLDLASESTAHAQSSK [252:284]
2987	1981	+3	111	28.2	18.1	23.0	1143.5094	3428.5136	3428.5248	-0.0112	0	×	LAEEEDLFDSAHPEEGDLDLASESTAHAQSSK
2988	1975	+4	62	7.9	7.3	5.5	857.8846	3428.5167	3428.5248	-0.0081	0	×	LAEEEDLFDSAHPEEGDLDLASESTAHAQSSK
2989	1970	+4	52	4.6	7.0	2.9	857.8866	3428.5246	3428.5248	-0.0003	0	×	LAEEEDLFDSAHPEEGDLDLASESTAHAQSSK
2990	1993	+3	112	31.5	20.4	32.0	1143.5144	3428.5287	3428.5248	0.0038	0	×	LAEEEDLFDSAHPEEGDLDLASESTAHAQSSK [253:284]
958	1754	+2	30	7.2	8.6	3.6	660.8489	1320.6906	1320.6906	-0.0000	0	√	NLWVSGLSSTTR [408:419]
1227	1768	+2	18	9.1	7.3	6.2	742.9079	1484.8085	1484.8108	-0.0022	2	×	ATDLKNLFSKYGK [420:432]
1918	2083	+3	75	14.5	10.8	9.1	647.0352	1939.0911	1939.1011	-0.0100	3	×	ATDLKNLFSKYGKVVGAK
1919	2073	+4	30	11.9	6.6	8.1	485.5294	1939.0957	1939.1011	-0.0054	3	×	ATDLKNLFSKYGKVVGAK
1920	2067	+4	33	15.4	7.3	8.9	485.5298	1939.0974	1939.1011	-0.0037	3	×	ATDLKNLFSKYGKVVGAK
1921	2078	+3	71	13.3	11.2	7.6	647.0383	1939.1003	1939.1011	-0.0009	3	×	ATDLKNLFSKYGKVVGAK [420:437]
52	280	+2	58	12.4	16.7	5.7	425.2302	849.4531	849.4577	-0.0047	0	√	HHSVEIK
53	285	+2	33	8.3	13.6	2.9	425.2313	849.4552	849.4577	-0.0025	0	√	HHSVEIK [511:517]
495	664	+2	35	10.7	10.2	6.9	522.3043	1043.6014	1043.6095	-0.0082	1	√	SKGEPVISVK
496	671	+2	33	12.6	9.5	5.2	522.3047	1043.6022	1043.6095	-0.0073	1	√	SKGEPVISVK [577:586]
550	1689	+2	58	9.6	14.0	6.0	539.8074	1078.6075	1078.6143	-0.0068	1	√	DILSFDKIK
551	1685	+2	52	10.9	14.0	4.3	539.8085	1078.6098	1078.6143	-0.0045	1	√	DILSFDKIK [610:618]
178	621	+2	57	15.9	14.3	10.5	451.2288	901.4504	901.4526	-0.0023	0	√	LEAFHER
179	615	+2	55	14.3	14.1	10.5	451.2291	901.4510	901.4526	-0.0017	0	√	LEAFHER [651:657]
571	554	+2	26	12.8	5.5	6.5	545.7874	1090.5674	1090.5752	-0.0078	2	×	RPYDLDRR
572	559	+2	23	12.2	4.6	6.5	545.7911	1090.5749	1090.5752	-0.0003	2	×	RPYDLDRR [730:737]
360	553	+2	40	10.3	12.0	6.9	487.2137	973.4202	973.4275	-0.0073	0	×	FHDFDHR
361	548	+2	48	10.3	12.8	6.9	487.2164	973.4256	973.4275	-0.0019	0	×	FHDFDHR [765:771]
811	508	+2	37	13.0	9.7	8.0	622.7776	1244.5480	1244.5555	-0.0075	1	×	FHDFDHRDR [765:773]

B.

HIT 10

Protein Mass: 102579.518 (monoisotopic) 102640.911(average) Protein Score: 389 Protein pp: 6503.2
sp|Q15424|SAFB1_HUMAN Scaffold attachment factor B1 OS=Homo sapiens GN=SAFB PE=1 SV=4

Sequence:

```
 001 MAETLSGLGD SGAAGAAALS SASSETGTRR LSDLRVIDLR AELRKRNVDS SGNKSVLMER LKKAIEDEGG NPDEIEITSE GNKKTSKRSS KGRKPEEEGV  100
 101 EDNGLEENSG DGQEDVETSL ENLQDIDIMD ISVLDEAEID NGSVADCVED DDADNLQESL SDSRELVEGE MKELPEQLQE HAIEDKETIN NLDTSSSDFT  200
 201 ILQEIEEPSL EPENEKILDI LGETCKSEPV KEESSELEQP FAQDTSSVGP DRKLAEEEDL FDSAHPEEGD LDLASESTAH AQSSKADSLL AVVKREPAEQ  300
 301 PGDGERTDCE PVGLEPAVEQ SSAASELAEA SSEELAEAPT EAPSPEARDS KEDGRKFDFD ACNEVPPAPK ESSTSEGADQ KMSSPEDDSD TKRLSKEEKG  400
 401 RSSCGRNFWV SGLSSTTRAT DLKNLFSKYG KVVGAKVVTN ARSPGARCYG FVTMSTAEEA TKCINHLHKT ELHGKMISVE KAKNEPVGKK TSDKRDSDGK  500
 501 KEKSSNSDRS TNLKRDDKCD RKDDAKKGDD GSGEKSKDQD DQKPGPSERS RATKSGSRGT ERTVVMDKSK GVPVISVKTS GSKERASKSQ DRKSASREKR  600
 601 SVVSFDKVKE PRKSRDSESH SRVRERSERE QRMQAQWERE ERERLEIARE RLAFQRQRLE RERMERERLE RERMHVEHER RREQERIHRE REELRRQQEL  700
 701 RYEQERRPAV RRPYDLDRRD DAYWPEAKRA ALDERYHSDF NRQDRFHDFD HRDRGRYPDH SVDRREGSRS MMGEREGQHY PERHGGPERH GRDSRDGWGG  800
 801 YGSDKRMSEG RGLPPPPRRD WGDHGRREDD RSWQGTADGG MMDRDHKRWQ GGERSMSGHS GPGHMMNRGG MSGRGSFAPG GASRGHPIPH GGMQGGFGGQ  900
 901 SRGSRPSDAR FTRRY                                                                                              915
```

Sequence Coverage: 16%
Sequence Tag Coverage: 13%

Index	scan#	charge	score	pp	pp_2	pp_{tag}	m/z	MW(obs)	MW	delta	miss	Unique	sequence + modifications	[start:end]
2283	1213	+3	62	4.7	8.2	3.2	749.0156	2245.0321	2245.0466	-0.0145	1	√	AIEDEGGNPDEIEITSEGNKK	[64:84]
2728	1592	+3	65	11.2	11.8	9.9	950.0995	2848.2841	2848.3119	-0.0278	1	×	SEPVKEESSELEQPFAQDTSSVGPDR	[227:252]
3040	1874	+4	101	17.0	11.9	12.0	889.9098	3556.6173	3556.6198	-0.0025	1	×	KLAEEEDLFDSAHPEEGDLDLASESTAHAQSSK	
3041	1883	+3	138	28.9	19.7	23.9	1186.2136	3556.6263	3556.6198	0.0065	1	×	KLAEEEDLFDSAHPEEGDLDLASESTAHAQSSK	[253:285]
2987	1981	+3	111	28.2	18.1	23.0	1143.5094	3428.5136	3428.5248	-0.0112	0	×	LAEEEDLFDSAHPEEGDLDLASESTAHAQSSK	
2988	1975	+4	62	7.9	7.3	5.5	857.8846	3428.5167	3428.5248	-0.0081	0	×	LAEEEDLFDSAHPEEGDLDLASESTAHAQSSK	
2989	1970	+4	52	4.6	7.0	2.9	857.8866	3428.5246	3428.5248	-0.0003	0	×	LAEEEDLFDSAHPEEGDLDLASESTAHAQSSK	
2990	1993	+3	112	31.5	20.4	32.0	1143.5144	3428.5287	3428.5248	0.0038	0	×	LAEEEDLFDSAHPEEGDLDLASESTAHAQSSK	[254:285]
1052	410	+2	63	14.0	12.1	8.8	684.2903	1367.5733	1367.5743	-0.0011	1	√	MSSPEDDSDTKR	
1053	407	+2	59	14.0	12.0	7.8	684.2908	1367.5743	1367.5743	-0.0001	1	√	MSSPEDDSDTKR	[382:393]
1227	1768	+2	18	9.1	7.3	6.2	742.9079	1484.8085	1484.8108	-0.0022	2	×	ATDLKNLFSKYGK	[419:431]
1918	2083	+3	75	14.5	10.8	9.1	647.0352	1939.0911	1939.1011	-0.0100	3	×	ATDLKNLFSKYGKVVGAK	
1919	2073	+4	30	11.9	6.6	8.1	485.5294	1939.0957	1939.1011	-0.0054	3	×	ATDLKNLFSKYGKVVGAK	
1920	2067	+4	33	15.4	7.3	8.9	485.5298	1939.0974	1939.1011	-0.0037	3	×	ATDLKNLFSKYGKVVGAK	
1921	2078	+3	71	13.3	11.2	7.6	647.0383	1939.1003	1939.1011	-0.0009	3	×	ATDLKNLFSKYGKVVGAK	[419:436]
1429	293	+2	62	9.7	11.0	5.6	793.8723	1586.7372	1586.7405	-0.0033	2	√	SKDQDDQKPGPSER	
1430	287	+2	82	16.3	12.9	9.6	793.8739	1586.7405	1586.7405	0.0000	2	√	SKDQDDQKPGPSER	[536:549]
571	554	+2	26	12.8	5.5	6.5	545.7874	1090.5674	1090.5752	-0.0078	2	×	RPYDLDRR	
572	559	+2	23	12.2	4.6	6.5	545.7911	1090.5749	1090.5752	-0.0003	2	×	RPYDLDRR	[712:719]
360	553	+2	40	10.3	12.0	6.9	487.2137	973.4202	973.4275	-0.0073	0	×	FHDFDHR	
361	548	+2	48	10.3	12.8	6.9	487.2164	973.4256	973.4275	-0.0019	0	×	FHDFDHR	[746:752]
811	508	+2	37	13.0	9.7	8.0	622.7776	1244.5480	1244.5555	-0.0075	1	×	FHDFDHRDR	[746:754]
1029	393	+3	34	18.4	7.3	6.3	453.2285	1357.6709	1357.6720	-0.0011	2	√	GRYPDHSVDRR	
1030	400	+3	15	7.1	6.2	4.7	453.2286	1357.6712	1357.6720	-0.0008	2	√	GRYPDHSVDRR	
1031	392	+2	22	13.5	4.2	4.0	679.3393	1357.6713	1357.6720	-0.0007	2	√	GRYPDHSVDRR	
1032	397	+2	20	8.7	4.0	3.4	679.3394	1357.6716	1357.6720	-0.0004	2	√	GRYPDHSVDRR	[755:765]
